# Neuritis of the Peripheral Nerves. — II

**Published:** 1891-05-23

**Authors:** J. Michell Clarke

**Affiliations:** Assistant Physician Bristol General Hospital


### Neuritis of the Peripheral Nerves.—II.

By J. Michell Clarke, M.A., M.B., M.R.C.P., Assistant Physician Bristol General Hospital.

### Gouty Neuritis of the Ulnar Nerve.

The patient, a shoemaker, aged 59, looking older, said that he awaked on Sunday morning, at three a.m., with shooting pains in right hand, passing to the inner side of the elbow, and from thence to the shoulder. The pains came on absolutely suddenly, still persisted (five days), and were increased by the least attempt to move the fingers. The hand was powerless. He had been subject to winter cough for many years; nine years ago had had a classical attack of gout in his right great toe, and twelve months since a second attack in the right hand. For six months previous to the present illness he had suffered pain in the right arm after working for some time. The hand lay in the position of rest, with the fingers slightly flexed at all joints and the thumb partially adducted. The thumb could be flexed and abducted fairly well, but could not be opposed to the little finger, and adduction was imperfect; the index could be a little extended and flexed; but the other fingers were almost powerless, the only movement remaining being a slight degree of extension, which, however, caused pain. The movements of the arm and forearm were normal. There was great tenderness to pressure over the ulnar nerve behind the internal condyle of the elbow. Anæsthesia, nearly complete, existed over the inner half of the lower two-thirds of the forearm, over the ulnar side of the hand, and over the whole of the little and ring fingers, both on the anterior and posterior surface, and also on the palmar surface of the tips of the index and middle fingers. Sensation was, further, slightly deficient on the anterior aspect of the radial side of the forearm, on the outer side of the palm and dorsum of the hand, and over the rest of the index and middle fingers. It was normal over all parts of the thumb. Under treatment for gout, in a week's time the loss of sensation was limited to the little and ring fingers and the ulnar side of the hand, both front and back. The paralysis rapidly disappeared, and the whole of the symptoms had cleared up in the course of four weeks.

### Neuritis of the Sciatics.

Many, if not the majority, of cases of sciatica are due to a neuritis of the nerve, and are not therefore true neuralgias. In the greater number of cases the neuritis is slight, and severe pain is the only symptom. If inquired for, however, one can very frequently obtain evidence of the existence of slight numbness, of "pins and needles," or other subjective sensations in the toes. In more severe cases there is a distinct loss of sensation, most commonly over the outer side of the thigh and leg, less commonly over the back of the thigh as well. In the latter case the small sciatic is also affected, either concurrently or by the inflammation spreading up the great sciatic. In the worst cases, besides the severe pains and areas of anæsthesia, the muscles supplied by the nerve become weak and flabby, or even undergo wasting. Lately attention has been particularly directed to a complication of this kind, which consists in paralysis with atrophy of the muscles supplied by the peroneal nerve.

The following case affords an instance of the affection of sensation :—

The patient was a miner, aged 44. The mine in which he worked was a wet one, and he often remained standing for some hours in water. Ten days before he came under observation he was suddenly seized whilst going to his work in the early morning with severe shooting pains in the left hip and back of the thigh. The great pain totally prevented him from walking or working. Shortly afterwards he had "pins and needles" in the toes, and a feeling of numbness in the feet and over the back of the thigh to the leg. He could assign no cause for the illness. With the exception of rheumatic pains in the right shoulder, he had always been healthy and temperate. He walked very lame with the aid of a stick. On examination, tender points were detected (1) at the left sciatic notch over the exit of the nerve; (2) just below and behind the head of the fibula (peroneal nerve); (3) about four inches downwards from the head of the fibula; and (4) over the heel. There was partial anæsthesia over the outer and posterior aspect of the left thigh, over the outer side of the knee and of the leg to about midway between the knee and ankle; also in slight degree over the sole of the foot. The temperature sense was normal. There was no muscular wasting, but electrical examination showed that the muscles supplied by the left peroneal nerve reacted less readily to the faradic, and more readily to the constant current, than those supplied by the right peroneal, and there was also slight qualitative change, the closing contracture occurring as readily at the anode as at the cathode. Under treatment the man was practically well in a little over three weeks. Defects in muscular nutrition, as indicated by wasting and by changes in the electrical reactions and sensory disturbances, are important in sciatica, as they indicate that we have to deal with an actual inflammation of the nerve, and that the course of the illness may be proportionately longer. More especially is this the case where muscular wasting occurs, for this, as said above, may in the most severe cases entail some amount of permanent damage to the muscles, and hence of interference with the movements of the limb, and in any case must take some time before complete recovery can ensue. When this complication occurs, the best guide as to the duration of the case will be afforded by the changes in the electrical reactions.

In the treatment of sciatica, whether there is neuritis or neuralgia, no other remedy gives such good results as the application of the constant currents. The milder cases are often cured after half-a-dozen sittings, sometimes after three or four, and the pain is speedily relieved and the duration shortened in the more severe ones. I have seen cases that have lasted for six months, and in which the muscles of the limb, though not atrophied in the proper sense of the term, have become flabby, weak, and small from disease, cured in three or four weeks. Daily applications should be made, for ten minutes at a time. The positive pole should be placed on the nerve at the exit from the sciatic notch, and the negative over its entrance into the popliteal space; or, with the positive pole in the same position, the negative is gently stroked from above downwards over the muscles of the back of the thigh and of the calf and outer side of the leg; or the two plans may be combined, each being carried out for five minutes. The current used should be, at first, that from 8-12 cells of an active Leclanché battery; later it may be gradually increased in strength. I think it is often advantageous, while the poles are applied over the nerve, to reverse the direction of the current several times by means of the reverser. It is hardly necessary to add that the skin must be well bathed in warm salt and water before the application of the poles. Immediately that the pain has been relieved, generally after the first few sittings, massage of the limbs can be borne, and should be used as well as the above-mentioned electrical treatment. At first the length of time during which massage is carried out should be short, then gradually increased. Internally, antipyrin, in twenty or thirty grain doses, is most useful for the relief of pain, especially for the nocturnal pains. If this is not effective, morphia may be required. Cod liver oil should be given throughout, and arsenic, quinine, and cannabis indica are often of service.

